# Are we overestimating proton minibeam therapy effectiveness through physical dose metrics?

**DOI:** 10.3389/fonc.2025.1682511

**Published:** 2025-12-11

**Authors:** Kaan Dere, Ahmet Cemal Usta, Reyhan Serra Kurnaz, Liyong Lin, Yong Yang, Murat Surucu, Serdar Charyyev

**Affiliations:** 1 Computational Sciences Minerva University, San Francisco, CA, United States; 2 Bahcesehir Koleji, Istanbul, Türkiye; 3 ENKA Okullari, Kocaeli, Türkiye; 4 Radiation Oncology Emory University, Atlanta, GA, United States; 5 Radiation Oncology Stanford University, Stanford, CA, United States

**Keywords:** pMBRT, SFRT, minibeams, PVDR, biological dose, GRID, protons, LET

## Abstract

**Purpose:**

The conventional implementation of proton spatially fractionated radiotherapy (SFRT) uses physical collimators with millimeter apertures to generate minibeams, creating alternating regions of high-dose peaks and low-dose valleys. Current evaluation of SFRT effectiveness predominantly relies on physical quantities, particularly the peak-to-valley dose ratio (PVDR). While high PVDR and low valley doses have been correlated with improved normal tissue sparing, this physical metric-based approach provides an incomplete picture of the treatment’s biological impact. In this work, we aim to quantify biological dose for proton minibeams created using physical collimators and critically evaluate the adequacy of physical (PVDR_PHYS_) as compared to biologically weighted PVDR (PVDR_BIOL_).

**Methods:**

Monte Carlo simulations using TOPAS were performed to model proton minibeam arrangements with 70 and 150 MeV monoenergetic beams, as well as a Spread-Out Bragg Peak (SOBP) configuration to represent clinically relevant dose delivery. We investigated the impact of multiple collimator configurations on PVDR: collimator thickness (6.35 cm), hole diameter (1–3 mm), center-to-center (c-t-c) distances (2, 3, 4 mm for 1 mm hole, 4, 6, 8 mm for 2 mm hole) and air gaps (5, 10, and 15 cm) between the collimator and water phantom. For each configuration, 3D dose and dose-averaged LET (LET_d_) distributions were scored in water phantom, which were subsequently used for RBE calculation using the McNamara model with α/β ratios of 3 or 10 Gy.

**Results:**

Our comprehensive analysis revealed significant differences between physical and biological PVDR metrics across various configurations. For both 70 and 150 MeV beams, PVDR_BIOL_ was consistently lower than PVDR_PHYS_ by 1-25% and 1-21%, respectively, with the most pronounced differences observed at shallow depths, smaller air gaps with larger hole diameter and c-t-c distances. Similar reductions (3.9%-26.5%) in PVDR_BIOL_ were observed for the SOBP configuration, with the specific pattern depending on the energy composition and weighting of constituent layers.

**Conclusions:**

The significant variations between PVDR_PHYS_ and PVDR_BIOL_ across different beam energies, depths, and collimator configurations demonstrate that conventional PVDR calculations based solely on physical dose may not fully represent the biological impact of proton SFRT. These findings highlight the importance of incorporating radiobiological considerations when evaluating and optimizing proton minibeam therapy, potentially leading to more biologically informed treatment planning approaches.

## 1 Introduction

Normal tissue toxicity remains the primary dose-limiting factor in radiation therapy, preventing the delivery of curative doses to challenging tumors such as large, radioresistant, or critically located malignancies. This fundamental constraint has driven decades of innovation in radiation delivery techniques, with spatially fractionated radiotherapy (SFRT) emerging as a promising approach to widen the therapeutic window by exploiting the superior tolerance of healthy tissues to spatially modulated versus uniform irradiation (1–5).

SFRT originated with Alban Köhler’s kilovoltage GRID therapy in 1909, which used perforated lead screens to minimize adverse radiation effects to the skin when irradiating deep-seated tumors (6, 7). More than two decades later, unaware of Köhler’s work, Dr. Liberson of U.S. Marine Hospitals used GRID therapy to treat small animals and inoperable tumors, similarly observing increased skin tolerance (8). These early investigations using perforations of different shapes in lead sheets primarily addressed the skin sparing challenges of orthovoltage treatments (9). With the advent of megavoltage radiotherapy and linear accelerators in the 1970s, skin sparing was no longer the primary concern, but new challenges emerged regarding normal tissue tolerance of organs like lung, brain, and intestines (10). This revived interest in GRID therapy, leading researchers to develop concepts compatible with megavoltage radiotherapy using specially designed Cerrobend grid matrices, successfully treating 71 patients with advanced bulky tumors using 6 MV and 25 MV photons (11). SFRT using protons represents a more recent development, beginning with micrometer-scale proton microbeam therapy (12, 13), followed by studies exploring 0.3-0.7 mm beam sizes termed proton minibeam radiation therapy (pMBRT). Prezado and Fois provided the initial proof-of-concept through Monte Carlo (MC) simulations (14), followed by experimental dosimetry studies using cyclotron-accelerated protons (15, 16) and demonstration of normal brain tissue sparing in animal models (17). Current pMBRT implementations utilize pencil beam scanning (PBS) with additional collimation to achieve millimeter-scale beamlets, as pencil beams alone produce approximately 1 cm beam sizes that require further collimation (typically using brass or tungsten plates) to maximize the dose-volume effect (18–21), employing different collimator designs [such as 3D-printed plastic plates (22)]. Beyond their material composition, collimators also differ in terms of beamlet patterning, ranging from 1D slits (19) to 2D hexagonal arrays (18). Other methods include magnetically focusing the beam to create minibeams (23–25).

Clinical effectiveness in SFRT is predominantly quantified using the peak-to-valley dose ratio (PVDR), defined as the ratio of high-dose peaks to low-dose valleys in the spatial dose distribution. Preclinical studies consistently demonstrate that higher PVDR values correlate with markedly improved normal tissue sparing (26, 27). For example, rat whole-brain pMBRT studies using 0.4 mm-wide minibeams with surface PVDR ≈ 15 achieved tolerances of single-fraction peak doses up to 58 Gy (~25 Gy mean dose) without detectable neurotoxicity, whereas conventional uniform 25 Gy proton beams caused severe brain injury (27). These remarkable tissue-sparing effects have generated significant clinical interest (28–30).

However, current SFRT evaluation relies on physical dose distributions and fails to account for biologically relevant factors that significantly influence therapeutic outcomes. For clinical practices, relative biological effectiveness (RBE) is assumed to be constant throughout the volume, with the value being equal to 1.1 relative to high-energy photons (31). However, it is well known that the RBE increases with linear energy transfer (LET); and the high LET is very characteristic of protons that have been degraded or scattered by collimators. As protons slow, their LET (and thus ionization density) rises, driving up RBE in the low−energy tail (32). Recent experimental studies (33) have documented LET increases of 3.5–7 keV/μm in collimated proton fields compared to open beams, with corresponding increases in RBE that could substantially alter the biological dose landscape. Ueno et al. (34) demonstrated that protons scattered off brass collimator edges exhibit higher dose averaged LET (LET_d_), while Nabha et al. measured significant LET gains near the Bragg peak when using aperture collimation (33). Smith and Hyer (35) confirmed that energy−specific collimation in PBS shifts the upper−percentile LET by a few keV/μm in trimmed beamlets, creating localized high−LET hotspots in the penumbra. These LET enhancements drive RBE increases that are not captured in conventional evaluation of pMBRT, potentially leading to systematic overestimation of normal tissue sparing when relying solely on physical dose metrics.

The clinical implications of this knowledge gap are profound. If physical PVDR (PVDR_PHYS_) overestimates the true biological effectiveness of spatial dose modulation, current treatment evaluation may systematically underestimate normal tissue complication probabilities, particularly for late-responding tissues with low α/β ratios that are most sensitive to RBE variations. To better interpret potential tissue-sparing effects, spatial dose modulation must be evaluated through a biological lens that accounts for variable RBE. No prior work has directly compared PVDR_PHYS_ to a biologically weighted analogue (PVDR_BIOL_) that incorporates LET−driven RBE variations documented in scattered or degraded proton fields. Incorporating RBE−weighted dose calculations, by validated biophysical models into proton minibeam evaluation will reveal the accuracy of PVDR_PHYS_ as a surrogate for therapeutic ratio and guide the development of biologically optimized treatment plans (36–38).

In this comprehensive MC simulation study, we aim to bridge the gap between physical and biological endpoints in pMBRT by (1): quantifying biological dose distributions using validated RBE models for millimeter-scale PBS minibeams shaped by physical collimators (2); directly comparing conventional PVDR_PHYS_ with biologically weighted PVDR_BIOL_ across a comprehensive range of beam energies and geometric configurations.

## 2 Materials and methods

**Figure 1:**
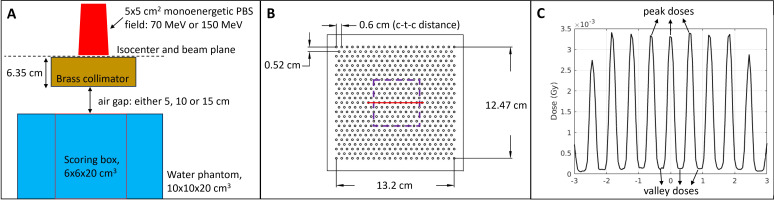
**(A)** Schematic diagram of the MC simulation geometry. Monoenergetic (70 MeV or 150 MeV) or SOBP proton beams with 5×5 cm² field size, created using an 11×11 spot array with 5 mm interspot spacing, pass through a 6.35 cm thick brass collimator positioned at variable air gaps (5, 10, or 15 cm) above a water phantom (10×10×20 cm³). Dose and LET_d_ scoring were performed within a central 6×6×20 cm³ region. **(B)** Top view of a representative hexagonal collimator pattern showing 2 mm diameter holes with 6 mm c-t-c spacing distributed across a 13.2×12.47 cm^2^ area. The purple dashed square delineates the 5×5 cm² proton field boundary, while the red dashed line indicates the lateral profile extraction path for PVDR analysis. **(C)** Representative lateral dose profile for 70 MeV proton beam at 1 cm depth demonstrating the characteristic peak-and-valley spatial dose modulation achieved through collimation. Peak doses correspond to beam paths through collimator apertures, while valley doses result from scattered radiation in inter-beam regions. This spatial fractionation pattern forms the basis for PVDR quantification across all geometric configurations studied.

MC simulations were performed using TOPAS (Tool for Particle Simulation) (39) version 3.7 with the default modular physics list without modification. The simulation geometry, **Figure 1A**, consisted of a 10×10×20 cm³ water phantom positioned below a brass collimator with variable air gaps. To ensure adequate statistical accuracy, 10^6^ particle histories per spot were simulated. The TOPAS simulation methodology employed in this study has been extensively validated through experimental benchmarking in our previous work. Charyyev et al. demonstrated good agreement between TOPAS simulations and film dosimetry measurements for similar minibeam collimator configurations, including the same collimator thickness, hole diameter, c-t-c distances, and air gaps used in the current study (40). Cross-validation with EBT3 film measurements showed agreement within 4.4% at the entrance region and 1% in the Bragg peak region across the energy range employed in this work (18). These validation studies provide robust experimental anchoring for the MC methodology used in the present investigation.

Monoenergetic proton beams of 70 MeV (continuous slowing down approximation (CSDA) range ~4.1 cm in water) and 150 MeV (CSDA range ~15.8 cm in water) were modeled with a 5×5 cm² field size created using an 11×11 spot array with 5 mm interspot spacing to generate a uniform incident field. The proton beam characteristics were modeled using realistic phase space parameters derived from clinical commissioning experimental measurements (41, 42). For 70 MeV protons, the beam was characterized by: spatial beam spread σ_x_ = σ_y_ = 5.74 mm, angular divergence σ_x_’ = σ_y_’ = 6.91 mrad, correlation coefficients ρ_x_ = ρ_y_ = 0.798, and energy spread = 1.36%. For 150 MeV protons: spatial beam spread σ_x_ = σ_y_ = 4.11 mm, angular divergence σ_x_’ = σ_y_’ = 3.47 mrad, correlation coefficients ρ_x_ = ρ_y_ = 0.625, and energy spread = 0.84%. These parameters were implemented using TOPAS emittance-based source modeling with BiGaussian distribution to accurately represent the correlated spatial-angular beam characteristics at the collimator entrance. Additionally, a Spread-Out Bragg Peak (SOBP) configuration was simulated to represent clinical dose delivery. The SOBP field consisted of five energy layers ranging from 100 to 110 MeV in increments of 2–3 MeV. Each energy layer was weighted according to the values shown in **Table 1** to produce a uniform dose plateau over depths of 7–9 cm in water. The primary analysis was based on a 2 Gy prescription dose, defined at the Bragg peak for pristine beams (or at the plateau for the SOBP configuration), Additional prescription dose levels of 5, 10 and 15 Gy were also investigated.

**Table 1:** Energy and relative weight of each energy used to obtain an SOBP.

Energy (MeV)	110	107	105	102	100
Relative weight	0.715	0.119	0.072	0.056	0.038

Brass collimator (density = 8.5 g/cm³, composition: 61.5% Cu, 35.5% Zn, and 3% Pb) with a thickness of 6.35 cm featured circular apertures arranged in hexagonal patterns with hole diameters of 1-, 2-, and 3-mm. **Figure 1B** illustrates a representative collimator design showing the hexagonal hole pattern for 2 mm diameter holes with 6 mm (0.6 cm) center-to-center (c-t-c) distance distributed across a 13.2×12.47 cm collimator area. The hexagonal arrangement was chosen as a motivation from Sammer et al. (43), who performed cell survival simulations for the various proton minibeam arrangements and showed that for a fixed tumor dose, the hexagonal beam arrangement provides the maximum benefits in normal tissue sparing. This geometric configuration spatially fractionated the initially uniform proton field into the characteristic minibeam pattern.

A comprehensive parameter study encompassed multiple configurations systematically varying the analyzed parameters for 70 MeV and 150 MeV: a) c-t-c distances scaled according to hole diameter: 1 mm holes: 2, 3, 4 mm c-t-c (corresponding to 2×, 3×, 4× hole diameter), 2 mm holes: 4, 6, 8 mm c-t-c, 3 mm holes: 6, 9, 12 mm c-t-c. b) air gap distances: 5, 10, and 15 cm between collimator exit surface and water phantom entrance, measured along the beam central axis (CAX).

The selection of proton energies, hole diameter, and c-t-c distances was based on clinical translation considerations and established optimization principles (18, 40, 43). The 70–150 MeV energy range represents clinically relevant energies for SFRT applications, with 70 and 150 MeV corresponding to approximately 4 and 16 cm range, respectively, in tissue, covering typical treatment depths for SFRT. Supra-millimeter hole diameters (1–3 mm) were selected based on practical optimization criteria that balance normal tissue sparing with clinical feasibility. While theoretically smaller apertures would enhance tissue sparing, 1–3 mm beamlets represent an optimal compromise considering several factors: beamlet width should not be smaller than setup uncertainties and beam delivery accuracy, cardiopulmonary motion effects on dose distribution, manufacturing feasibility compared to micrometric apertures, achievable dose rates and treatment times, beam economy with reduced collimator activation, and avoidance of excessively high entrance doses that can exceed Bragg peak values due to reduced scatter and loss of charged particle equilibrium. The c-t-c distances were determined to achieve uniform target dose coverage, with hexagonal arrangements requiring a σ_depth_/c-t-c distance ratio of approximately 0.40-0.44 for dose uniformity within 95-105% based on inverse planning studies (40, 43).

3D dose and LET_d_ distributions were scored within a central 6×6×20 cm³ scoring region of the water phantom to focus analysis on the primary beam interaction volume while reducing computational overhead (**Figure 1A**). This scoring volume was positioned to capture the complete minibeam pattern. Dose and LET_d_ distributions were scored simultaneously throughout the scoring volume using a uniform voxel grid of 0.5×0.5×1.0 mm³ and 1.0×1.0×10.0 mm³, respectively. LET_d_ used larger scoring voxels to prevent numerical singularities in the LET calculation arising from low-dose regions. LET_d_ calculations included contributions from primary protons and secondary protons only, including the energy deposited by associated secondary electrons but excluding heavier fragments (alpha particles, nuclear fragments), which contribute<0.1% of total dose, to maintain consistency with established clinical RBE models. LET_d_ distributions were interpolated from the coarser scoring grid to match the finer dose scoring grid using trilinear interpolation.

The collimator design successfully generated the intended spatially fractionated dose distribution, as demonstrated in **Figure 1C**, which shows a representative lateral dose profile for 70 MeV minibeams at 1 cm depth displaying the characteristic peak and valley pattern. Peak doses correspond to regions aligned with collimator apertures, while valley doses represent the inter-beam regions where only scattered radiation contributes to the dose.

RBE was calculated using the McNamara (Equations 1) (44) phenomenological model:

(1) $$\begin{array}{c} RBE\left[D,\left(\frac{\alpha }{\beta }\right),LET_{d}\right]\\ =\left(\frac{1}{2D}\right)\left(\sqrt{{\left(\frac{\alpha }{\beta }\right)}^{2}+4D\left(\frac{\alpha }{\beta }\right)\left(p_{0}+\frac{p_{1}}{\left(\frac{\alpha }{\beta }\right)}LET_{d}\right)+4D^{2}{\left(p_{2}+p_{3}\sqrt{\left(\frac{\alpha }{\beta }\right)}LET_{d}\right)}^{2}}-\left(\frac{\alpha }{\beta }\right)\right) \end{array}$$

where, D is physical dose, $\frac{\alpha }{\beta }$ represents intrinsic radiosensitivity, $LET_{d}$ is dose averaged LET, $p_{0}=0.99064,\;p_{1}=0.35605,p_{2}=1.1012\;and\;p_{3}=-0.0038703.$This model was applied on a voxel-by-voxel basis using the local physical dose and corresponding LET_d_ values. Two different α/β ratios of 3 Gy and 10 Gy were employed to represent different tissue response characteristics, with the biological dose calculated as $D_{BIOL}=D\times RBE$ for each voxel. The McNamara model was selected due to its comprehensive validation against experimental data and its widespread use in proton therapy research (31, 45–48). However, we have done calculations for selected subgroup of simulation scenarios for two other RBE models: Carabe-Fernandez (Equations 2–4) (49):

(2) $$RBE\left[D,\left(\frac{\alpha }{\beta }\right),LET_{d}\right]=\frac{1}{2D}\left(\sqrt{{\left(\frac{\alpha }{\beta }\right)}^{2}+4D\left(\frac{\alpha }{\beta }\right)RBE_{max}+4RBE_{min}^{2}D^{2}}-\left(\frac{\alpha }{\beta }\right)\right)$$

where, D is physical dose, $\frac{\alpha }{\beta }$ represents intrinsic radiosensitivity, $LET_{d}$ is dose averaged LET, $RBE_{max}$ and $RBE_{min}$ (derived using V79 cell data) are defined as follows:

(3) $$RBE_{max}\left[LET_{d},\left(\frac{\alpha }{\beta }\right)\right]=0.843+0.154\frac{2.686}{\left(\frac{\alpha }{\beta }\right)}LET_{d}$$

(4) $$RBE_{min}\left[LET_{d},\left(\frac{\alpha }{\beta }\right)\right]=1.09+0.006\frac{2.686}{\left(\frac{\alpha }{\beta }\right)}LET_{d}$$

and Wedenberg (Equation 5) (50):

(5) $$RBE\left[D,\left(\frac{\alpha }{\beta }\right),LET_{d}\right]=\frac{1}{2D}\left(\sqrt{{\left(\frac{\alpha }{\beta }\right)}^{2}+4D\left(\left(\frac{\alpha }{\beta }\right)+0.434LET_{d}\right)+4D^{2}}-\left(\frac{\alpha }{\beta }\right)\right)$$

where, D is physical dose, $\frac{\alpha }{\beta }$ represents intrinsic radiosensitivity, $LET_{d}$ is dose averaged LET.

PVDR analysis was performed using lateral dose profiles, similar to that in **Figure 1C**, extracted perpendicular to the minibeam arrangement through the field center. Lateral profiles were sampled at dose resolution to ensure accurate determination of peak and valley positions, with analysis performed at multiple depths from the phantom surface to near-maximum beam range.

Physical PVDR was calculated as the ratio of average peak doses to average valley doses from the physical dose profiles: $PVDR_{PHYS}=\frac{Average\;peak\;doses}{Average\;valley\;doses}$. Similarly, biological PVDR was determined using RBE-weighted dose profiles: $PVDR_{BIOL}=\frac{Average\;RBE-weighted\;peak\;doses}{Average\;RBE-weighted\;valley\;doses}$. To minimize edge effects, the analysis was restricted to the central region of the field, excluding peaks and valleys within 1 cm of the field boundary. The PVDR calculations were systematically performed across all parameter combinations, including different hole sizes, c-t-c distances, air gaps, and beam energies, enabling comprehensive evaluation of the relationship between physical and biological dose metrics in pBMRT.

Voxel-wise uncertainties in physical dose and LET_d_ were scored in TOPAS and propagated through all calculations using standard error propagation. For PVDR_PHYS_, uncertainties in peak and valley doses were combined in quadrature using standard ratio error propagation. For PVDR_BIOL_, the uncertainty calculation required additional steps due to RBE’s dependence on both dose and LET_d_. Partial derivatives of the McNamara RBE model with respect to dose and LET_d_ were calculated analytically to determine RBE uncertainty at both peak and valley locations. These RBE uncertainties were then combined with physical dose uncertainties to calculate biological dose uncertainties. Finally, PVDR_BIOL_ uncertainty was determined by propagating the biological dose uncertainties through the PVDR calculation.

Simulations were performed on Intel^®^ Xeon(R) Gold system with parallel processing utilizing 96 CPU cores per simulation. Total computational time was approximately 110 hours for the complete parameter study.

## 3 Results

**Figure 2:**
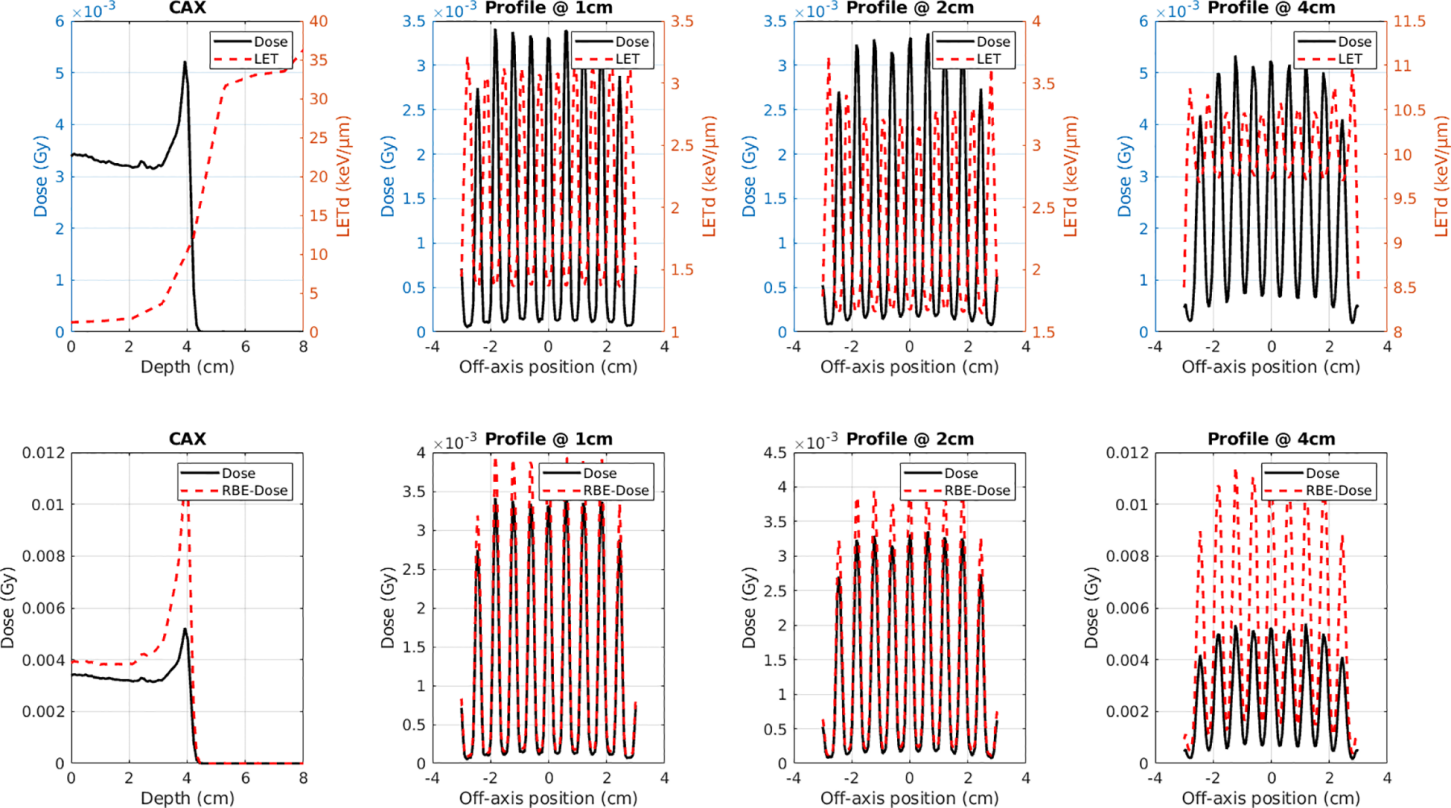
Representative dose and LET_d_ distribution analysis for 70 MeV proton SFRT with 5 cm air gap, 2 mm hole diameter, and 6 mm c-t-c distance. Top row: Physical dose (black solid) and LET_d_ (red dashed) distributions showing CAX depth profile and lateral profiles at 1, 2, and 4 cm depths. Bottom row: Corresponding physical dose (black solid) and RBE-weighted biological dose (red dashed) distributions. The lateral profiles clearly demonstrate the spatially fractionated beam delivery pattern with distinct peaks and valleys. This analysis was performed for all simulation scenarios in the study.

**Figure 2** shows a representative example of the dose and LET_d_ analysis performed for all simulation scenarios, specifically for the configuration with 5 cm air gap, 2 mm hole diameter, and 6 mm c-t-c distance for 70 MeV protons. The top row demonstrates the physical dose (black solid line) and LET_d_ (red dashed line) distributions, including the CAX depth profile and lateral profiles at 1, 2, and 4 cm depths. The bottom row presents the corresponding physical dose (black solid line) and RBE-weighted biological dose (red dashed line) distributions at the same positions. The lateral profiles clearly demonstrate the spatially fractionated nature of the beam delivery with distinct peaks and valleys. The LET_d_ distribution shows elevated values in the valley regions, contributing to enhanced biological effectiveness in these areas. The biological dose distributions consistently show higher values compared to physical dose, particularly in the valley regions where the combination of moderate dose levels and elevated LET_d_ results in significant RBE enhancement.

**Figure 3:**
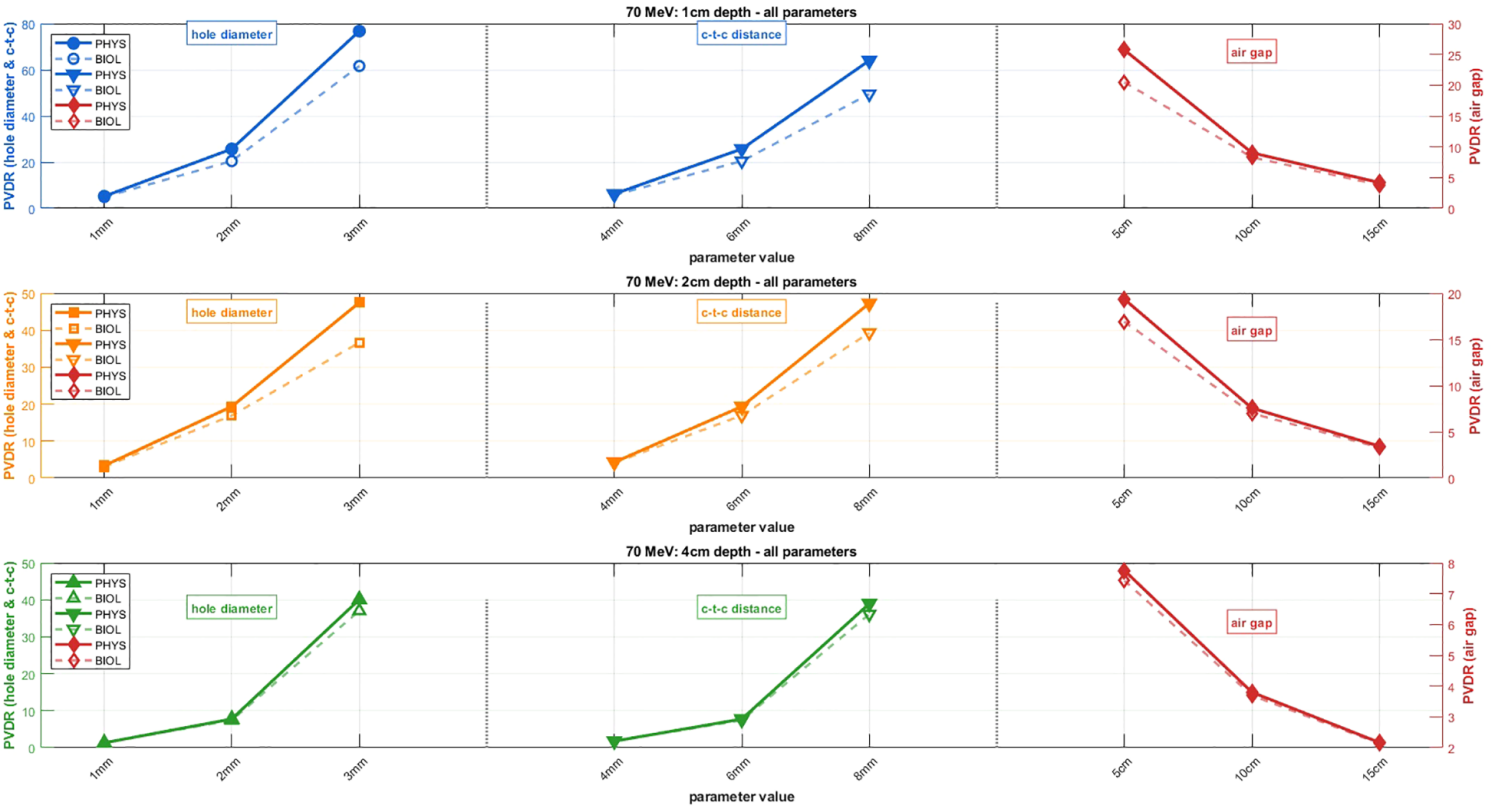
Parametric analysis of PVDR for 70 MeV proton beams showing both PVDR_PHYS_ (solid lines) and PVDR_BIOL_ (dashed lines) values. Rows represent different depths: 1 cm, 2 cm, and 4 cm. Parameter variations: hole diameter (1–3 mm with c-t-c distance = 3× hole diameter, air gap = 5 cm), c-t-c distance (4–8 mm with hole diameter = 2 mm, air gap = 5 cm), and air gap (5–15 cm with hole diameter = 2 mm, c-t-c distance = 6 mm).

**Figure 4:**
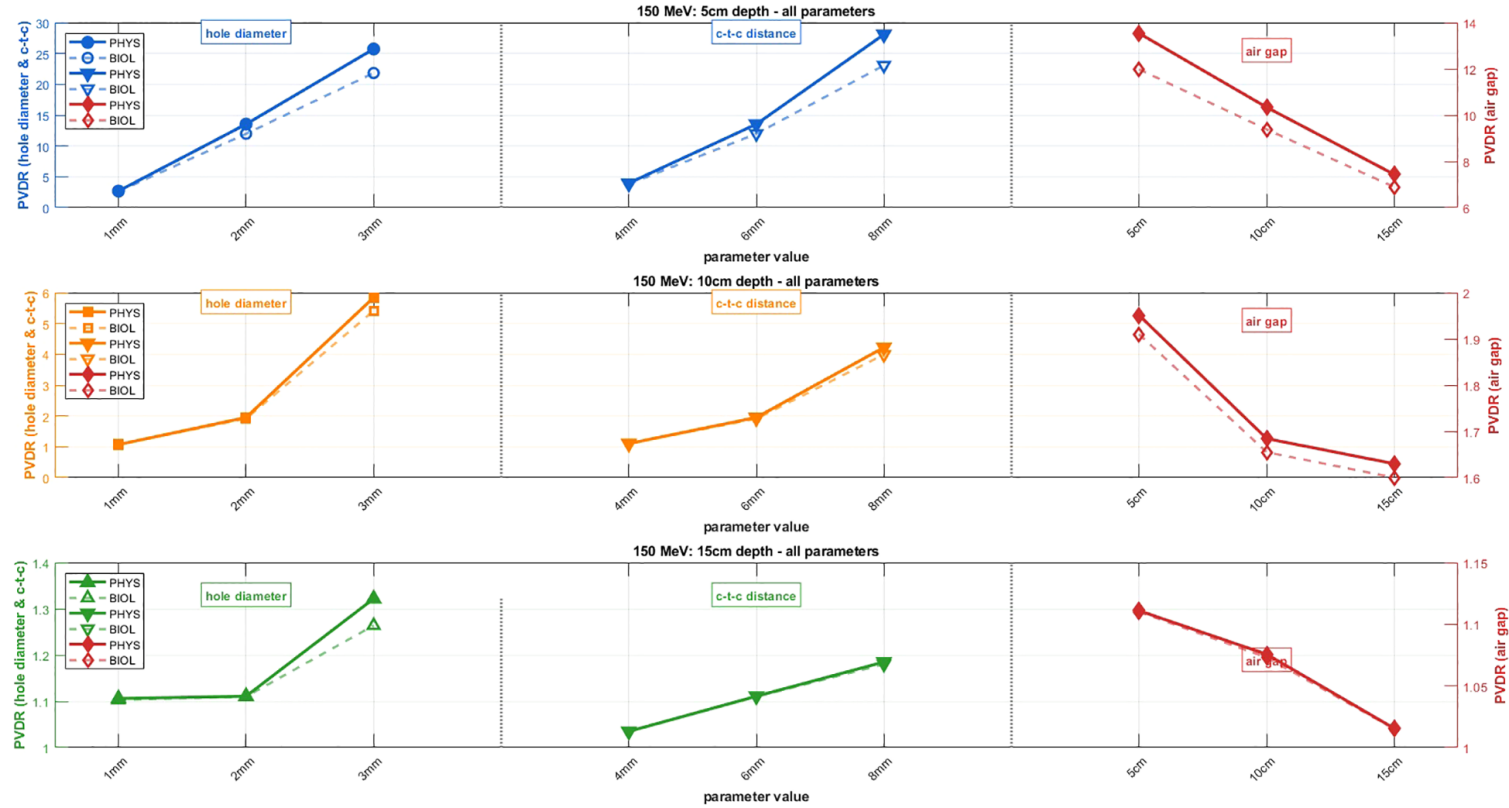
Parametric analysis of PVDR for 150 MeV proton beams with identical parameter variations as **Figure 3**. Rows represent depths: 5 cm, 10 cm, and 15 cm.

**Figures 3**, **4** present systematic parametric analyses of how PVDR values vary with geometric beam parameters for 70 MeV and 150 MeV protons, respectively. Both PVDR_PHYS_ (solid lines) and PVDR_BIOL_ (dashed lines) using the McNamara model are shown across three parameter variations. The relative uncertainties (σ/value) were less than 0.02% for PVDR_PHYS_ at both beam energies. For PVDR_BIOL_, relative uncertainties ranged from 1.1% to 2.9% at 70 MeV and from 0.7% to 3.3% at 150 MeV. Due to the small magnitude of the uncertainties relative to the marker size, uncertainty bars are not displayed in figures.

When varying hole diameter from 1 mm to 3 mm (with air gap fixed at 5 cm and c-t-c distance maintained at 3× hole diameter), both energies show substantial increases in PVDR. For 70 MeV at 1 cm depth, PVDR increases from approximately 5 to 80 for physical dose, while PVDR_BIOL_ shows a similar trend but with consistently lower absolute values. The 150 MeV configuration demonstrates more modest PVDR increases but follows the same trend, with values ranging from 2 to 25 at 5 cm depth. Variation of c-t-c distance (with 5 cm air gap and 2 mm hole diameter fixed) shows the strongest influence on PVDR among all tested parameters. For 70 MeV beams, increasing c-t-c distance from 4 mm to 8 mm results in dramatic PVDR increases from approximately 5 to 65 at 1 cm depth for physical dose. The 150 MeV configuration shows similar trends with more moderate absolute values.

The air gap parameter exhibits an inverse relationship with PVDR across both energies. For 70 MeV beams (with 2 mm hole diameter and 6 mm c-t-c distance), increasing air gap from 5 cm to 15 cm reduces PVDR from approximately 25 to 16 at 1 cm depth. This inverse relationship suggests that minimal air gaps are preferred for maximizing PVDR in proton SFRT configurations. The analysis reveals distinct depth-dependent behavior for both energies. For 70 MeV, depths of 1, 2, and 4 cm show progressively decreasing PVDR values across all parameter combinations. Similarly, for 150 MeV at depths of 5, 10, and 15 cm, PVDR values decrease with increasing depth, though the 150 MeV configuration maintains more consistent PVDR values across the analyzed depth range.

**Figure 5:**
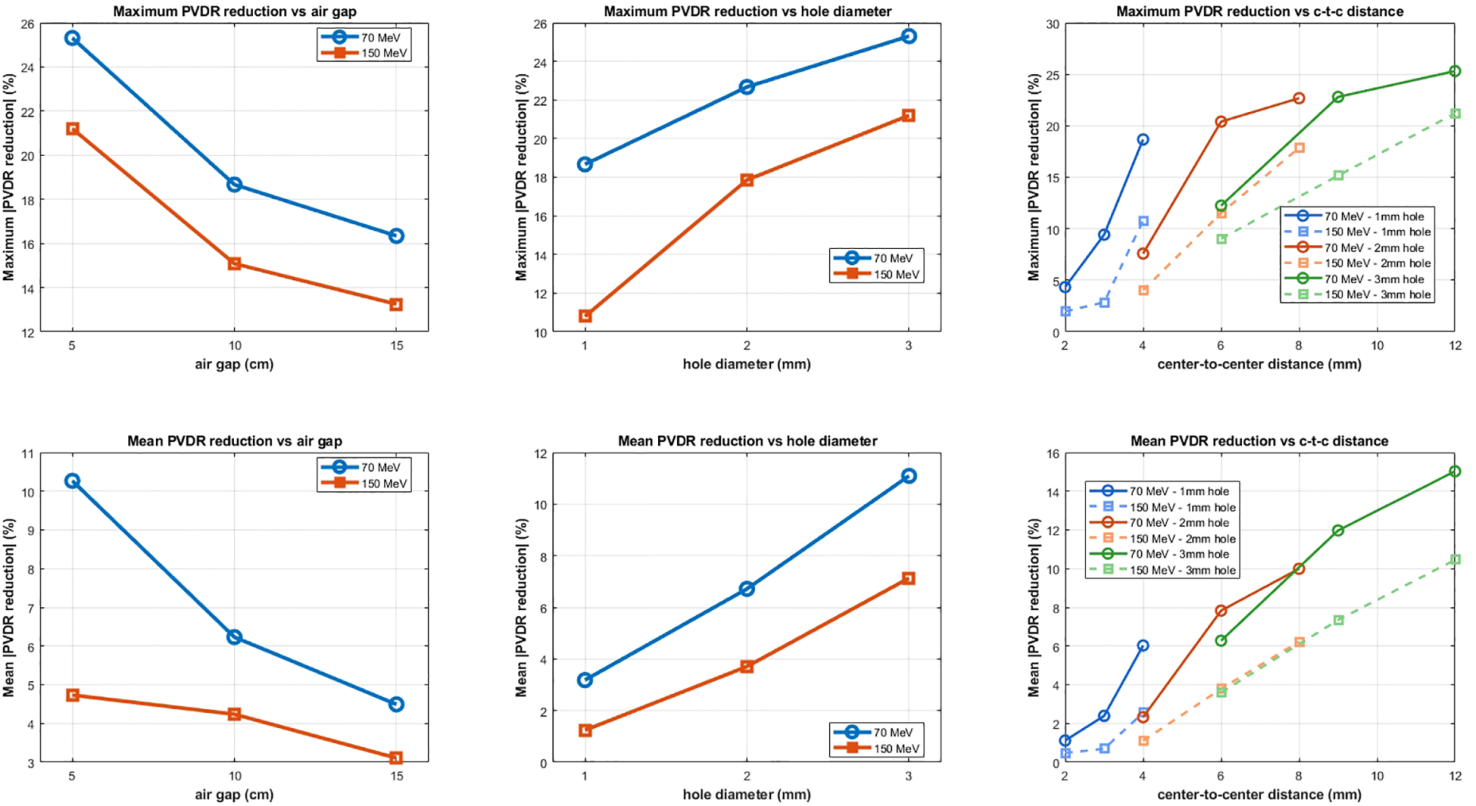
Quantitative comparison of PVDR_PHYS_ versus PVDR_BIOL_ showing maximum and mean PVDR reduction percentages across all parameter combinations. Top row: Maximum PVDR reduction for 70 MeV (blue) and 150 MeV (orange) as functions of air gap, hole diameter, and c-t-c distance. Bottom row: Corresponding mean PVDR reductions. The analysis demonstrates that PVDR_BIOL_ is consistently lower than PVDR_PHYS_, with the magnitude of reduction varying significantly based on beam parameters and energy. Multiple hole diameter configurations are shown for c-t-c distance analysis.

**Figure 5** provides a quantitative analysis of the differences between PVDR_PHYS_ and PVDR_BIOL_ across all parameter combinations and energies. The results demonstrate that PVDR_BIOL_ is consistently lower than PVDR_PHYS_, with the magnitude of reduction depending on the specific parameter combination and energy. For maximum PVDR reduction analysis, the 70 MeV configuration shows reductions of up to 25% for optimal parameter combinations, while 150 MeV shows more modest reductions of 10-20%. The mean PVDR reduction analysis reveals similar trends with slightly lower absolute values. The c-t-c distance parameter shows the most dramatic differences between PVDR_PHYS_ and PVDR_BIOL_, while air gap variations show the smallest difference. This occurs because increased c-t-c distance forces protons to traverse significantly more material to reach valley regions, reducing their energy and increasing their LET_d_ (and thus RBE), creating larger disparities between PVDR_PHYS_ and PVDR_BIOL_. Conversely, air-gap variations primarily affect beam divergence and penumbra without substantially altering proton energies, resulting in smaller PVDR differences.

**Figure 6:**
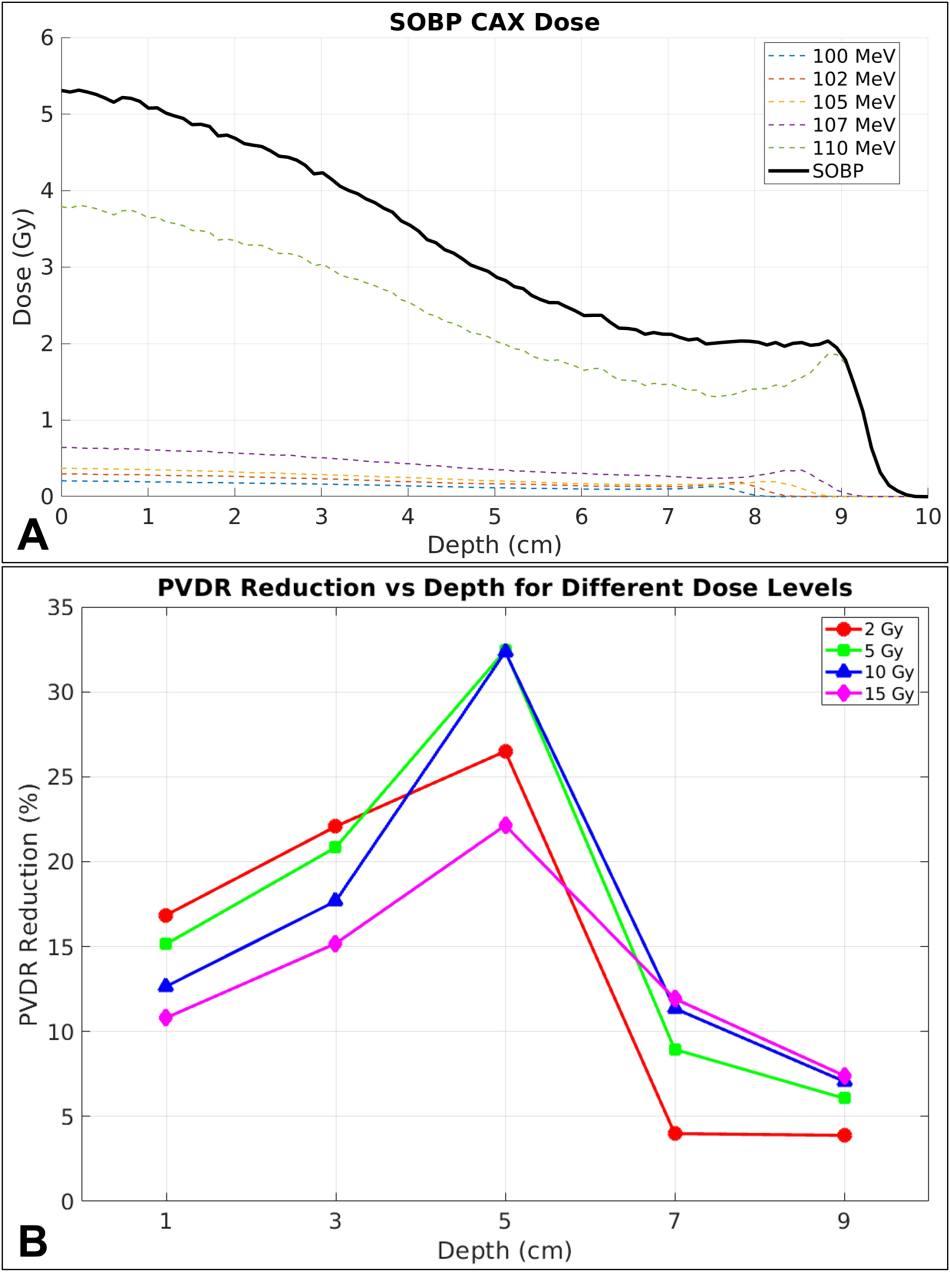
**(A)** CAX depth dose profiles showing individual energy layer contributions (dashed lines) and the resulting SOBP (solid black line) achieving a uniform dose plateau over 7–9 cm depth. **(B)** PVDR reduction as a function of depth for different dose levels using the McNamara model with α/β = 3 Gy. All calculations performed with 2 mm hole diameter, 6 mm c-t-c distance, and 5 cm air gap.

**Figure 6A** illustrates the depth dose profiles of the individual energy layers (100, 102, 105, 107, and 110 MeV) and their weighted superposition forming the SOBP. **Figure 6B** demonstrates the PVDR reduction as a function of depth for different prescription dose levels (2, 5, 10, and 15 Gy) using the McNamara model with α/β = 3 Gy. The prescription level was determined by the dose delivered to the SOBP plateau, with the 2 Gy example shown in **Figure 6A**. PVDR reduction ranging from 3.9% to 26.5% (for the 2 Gy prescription level) is evident with the SOBP scenario as well, with the specific pattern depending on the energy composition and weighting of constituent layers.

**Figure 7:**
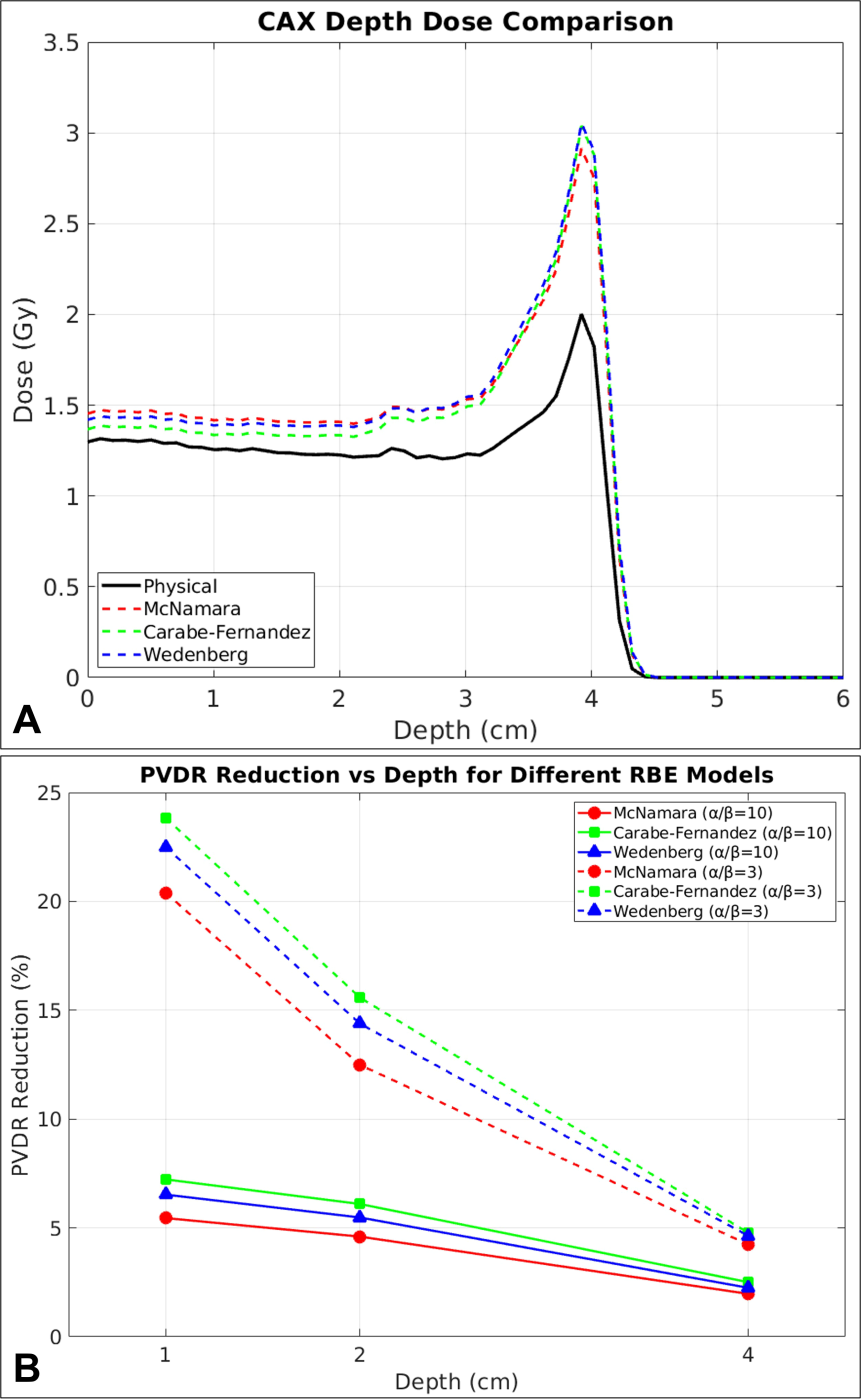
**(A)** CAX depth dose profiles showing physical dose (black solid line) and RBE-weighted doses calculated using McNamara (red dashed), Carabe-Fernandez (green dashed), and Wedenberg (blue dashed) models. **(B)** PVDR reduction comparison between different tissue radiosensitivities (α/β ratios) for all three RBE models. Solid lines represent α/β = 10 Gy (more radioresistant tissues) while dashed lines represent α/β = 3 Gy (more radiosensitive tissues). McNamara model (red circles), Carabe-Fernandez model (green squares), and Wedenberg model (blue triangles) demonstrate consistent trends with significantly higher PVDR reduction for α/β = 3 Gy compared to α/β = 10 Gy.

**Figure 7A** demonstrates the comparative analysis of dose distributions calculated using three different RBE models: McNamara (44), Carabe-Fernandez (49), and Wedenberg (50). The CAX depth dose comparison reveals that all three RBE models produce enhanced dose delivery compared to physical dose calculations, with the enhancement becoming more pronounced at greater depths where LET values increase substantially.

**Figure 7B** illustrates the comparative PVDR reduction analysis for both α/β = 3 Gy and α/β = 10 Gy across all three RBE models as a function of depth. The results demonstrate the substantial impact of tissue radiosensitivity on biological dose calculations. For α/β = 3 Gy, representing more radiosensitive tissues, PVDR reductions range from approximately 20-24% at 1 cm depth to 4-5% at 4 cm depth across all models. In contrast, for α/β = 10 Gy, representing more radioresistant tissues typically associated with early-responding tissues and tumors, the reductions are substantially lower, ranging from 5 to 7% at 1 cm depth to 2-3% at 4 cm depth.

## 4 Discussion

Among the results presented in the Results section, several key observations warrant detailed discussion. The clinical significance of the observed 20-25% reduction in PVDR must be evaluated within the context of current understanding of SFRT effectiveness, a complex undertaking in itself (51). Typical pMBRT PVDR values achieved in clinical practice range from 9-12 (52, 53). However, the field currently lacks definitive evidence establishing optimal PVDR thresholds for different anatomical sites and clinical scenarios. The NRG Oncology/AAPM Working Group consensus (30) acknowledges that PVDR parameters remain “unclear” and require further clinical validation, though general agreement exists that spatial dose modulation must be sufficient to preserve normal tissue sparing mechanisms. Despite this uncertainty in optimal PVDR values, a 20-25% reduction in PVDR represents a clinically significant finding that cannot be dismissed. Preclinical evidence demonstrates that PVDR operates within narrow therapeutic windows, with mathematical models showing therapeutic ratios varying dramatically (0.88 to 13.22) depending on achieved PVDR values (54). In the absence of established PVDR thresholds, treatment planning systems that rely solely on physical dose calculations may systematically overestimate the therapeutic benefit of proton SFRT, particularly for treatments involving late-responding critical structures where the biological dose reduction is most pronounced.

Our findings are strongly supported by the pioneering work of Meyer et al. (55), who first implemented RBE-weighted dose calculations in pMBRT using TOPAS Monte Carlo simulations and the MC damage simulation model for double strand break endpoints. Despite methodological differences—Meyer et al. used 300 μm steel slits with 1 mm center-to-center spacing while our study employed 1–3 mm circular brass apertures with 2-4× hole diameter spacing—both studies demonstrate remarkably consistent quantitative results. Meyer et al. reported that relative mean RBE-weighted entrance dose was approximately 25% lower than physical dose for proton minibeams, which closely aligns with our finding that PVDR_BIOL_ decreases by 20-25% compared to PVDR_PHYS_ for late-responding tissues (α/β = 3 Gy). Both studies also confirm similar depth-dependent RBE variations, with entrance RBE values around 1.02-1.04, Bragg peak RBE of 1.3-1.6, and distal edge values reaching 1.4-2.0. This convergent evidence from independent methodological approaches provides robust validation that biological dose effects significantly impact proton SFRT dosimetry. Our work extends Meyer’s foundation by systematically examining how RBE affects the fundamental PVDR parameter that defines SFRT’s therapeutic advantage, while providing multi-model validation through multiple phenomenological RBE approaches and comprehensive geometric parameter analysis across two proton energies and SOBP configurations.

An important observation from our SOBP analysis is that the resulting depth-dose distribution does not resemble the classical flat plateau achieved with conventional proton PBS. This reflects a unique characteristic of proton minibeam depth-dose curves: the peak dose decreases rapidly with depth such that the maximum dose occurs at the entrance rather than in the target region, unlike conventional PBS proton therapy. As shown in **Figure 6A**, the SOBP dose reaches only approximately 40% of the entrance peak dose for 2 mm minibeams, an effect that becomes more prominent as minibeam diameter decreases. While this characteristic may initially appear undesirable for normal tissue sparing, it is important to recognize that these peak dose regions exist at very small scales of ~2–3 mm and occupy only a small fraction (~10%) of the normal tissue volume. The bulk of the normal tissue (~90%) receives substantially lower valley doses.

Our choice of the McNamara model for primary RBE calculations was based on its widespread use and established popularity in the proton therapy community. However, clinicians and researchers may choose alternative RBE models depending on their goals and preferences. From our analysis, at the shallower depths McNamara model produced the highest dose enhancement, followed by the Wedenberg model, while the Carabe-Fernandez model provided the most conservative estimates. This ordering reversed at the Bragg peak depth, where the Carabe-Fernandez model produced the highest dose enhancement, followed by Wedenberg, while the McNamara model provided the most conservative estimates.

It should be noted that any chosen RBE model, including the ones we selected for this study, has inherent uncertainties and that patient-specific radiosensitivities may be different. Also, the RBE models used in this study are based on cell survival models but don’t consider other biological endpoints like vascular effects, immune responses, or repair mechanisms that might be particularly relevant for SFRT. Furthermore, current RBE models face additional limitations in hypo-fractionated SFRT conditions, as they were developed using conventional fractionation data. Even though we demonstrated PVDR reduction for different prescription levels, **Figure 6B**, the underlying linear-quadratic model systematically fails at high doses per fraction (>8–10 Gy) characteristic of SFRT peak regions, where vascular damage and immune activation mechanisms become dominant contributors beyond DNA damage-based calculations (56, 57). Therefore, our PVDR_BIOL_ calculations represent first-order approximations that should be interpreted with appropriate caution in the context of these model limitations. These limitations underscore the critical need for experimental validation of RBE models under hypo-fractionated SFRT conditions (31).

The choice of α/β = 3 Gy for our primary analysis was made for several clinical reasons. First, α/β = 3 Gy represents late-responding normal tissues such as spinal cord, brain, and lung tissue, which are typically the dose-limiting structures in radiotherapy planning. For normal tissue sparing evaluation, α/β = 3 Gy provides a more conservative and clinically relevant estimate of biological effects. Second, in SFRT, the primary clinical advantage lies in sparing late-responding normal tissues, and most critical organs at risk have lower α/β ratios rather than higher. Finally, this choice aligns with literature precedent, as many proton RBE studies focusing on normal tissue effects utilize α/β ratios of 2–3 Gy. However, it is important to acknowledge that different clinical scenarios may warrant different α/β ratio selections. The comparison in **Figure 7** emphasizes that the choice of α/β ratio significantly influences the magnitude of biological effects in proton SFRT, and treatment planning should consider the specific tissue types being treated and their associated radiobiological parameters.

Although extensive simulations were performed across multiple parameter combinations, we showcased the specific case of 2 mm hole diameter with 6 mm c-t-c distance and 5 cm air gap for several reasons. We believe that millimeter-sized proton beams are preferred over sub-millimeter proton beams because sub-millimeter beams cannot maintain high PVDRs at larger depths in patients due to lateral scattering effects. For this reason, peak-to-valley contrast even for 1 mm holes is not well pronounced, especially for 150 MeV protons as they travel deeper into tissue. Since PVDR values around 10 have been shown to produce minimal damage to normal tissue, a PVDR of 10 at the phantom surface was targeted as the minimum quality threshold for the selected minibeams. This target PVDR value was achieved with c-t-c distances of at least 6 mm for 2 mm hole diameter configurations. From a practical clinical standpoint, larger air gaps are generally preferred as the additional space may be needed for positioning and imaging systems. However, due to scattering effects in air, larger air gaps result in poorer PVDR values. Therefore, the smallest possible and clinically safe air gap should be considered for clinical implementation.

While this study establishes differences between PVDR_PHYS_ and PVDR_BIOL_, future work is needed to determine clinical adequacy thresholds and develop specific treatment planning recommendations.

## 5 Conclusion

This comprehensive MC study demonstrates that biological dose calculations alter the dosimetric landscape of pMBRT compared to conventional physical dose planning. Through systematic analysis of geometric configurations across two proton energies and SOBP configuration, we have shown that incorporating RBE effects using established phenomenological models results in notable reductions in PVDRs, with PVDR_BIOL_ decreasing by up to 26% compared to physical calculations for late-responding normal tissues (α/β = 3 Gy). While the clinical field currently lacks consensus on optimal PVDR thresholds, the magnitude of these reductions cannot be dismissed given the narrow therapeutic windows demonstrated in preclinical studies and the documented sensitivity of late-responding tissues to spatial dose modulation patterns. Our findings reveal that current treatment planning approaches relying solely on physical dose calculations may systematically overestimate the therapeutic benefit of proton SFRT, particularly for treatments involving critical structures with low α/β ratios where biological dose enhancement is most pronounced. The observed parametric dependencies, with c-t-c distance showing the strongest influence on PVDR across all models, remain consistent between physical and biological calculations, though the absolute PVDR values are consistently reduced when biological effects are considered. These results underscore the critical importance of incorporating RBE modeling into proton SFRT treatment planning systems and highlight the need for experimental validation of PVDR thresholds to optimize the clinical implementation of this promising therapeutic approach.

## Data Availability

The original contributions presented in the study are included in the article/supplementary material. Further inquiries can be directed to the corresponding author.
